# Emerging role of interaction between m6A and main ncRNAs in gastrointestinal (GI) cancers

**DOI:** 10.3389/fimmu.2023.1129298

**Published:** 2023-02-17

**Authors:** Yating Xu, Xiao Yu, Wenzhi Guo, Yuting He

**Affiliations:** ^1^ Department of Hepatobiliary and Pancreatic Surgery, The First Affiliated Hospital of Zhengzhou University, Zhengzhou, China; ^2^ Key Laboratory of Hepatobiliary and Pancreatic Surgery and Digestive Organ Transplantation of Henan Province, The First Affiliated Hospital of Zhengzhou University, Zhengzhou, China; ^3^ Open and Key Laboratory of Hepatobiliary & Pancreatic Surgery and Digestive Organ Transplantation at Henan Universities, Zhengzhou, China; ^4^ Henan Key Laboratory of Digestive Organ Transplantation, The First Affiliated Hospital of Zhengzhou University, Zhengzhou, China

**Keywords:** gastrointestinal cancers, m6A, lncRNA, miRNA, circRNA

## Abstract

As a prevalent epigenetic modification, the role of m6A has been increasingly highlighted in the alteration of numerous RNAs implicated with multiple biological processes, such as formation, export, translation, and degradation. With further the understanding of m6A, accumulating evidence shows that m6A modification similarly affects metabolic process of non-coding genes. But the specifical interplay of m6A and ncRNAs (non-coding RNAs) in gastrointestinal cancers still lacks complete discussion. Thus, we analyzed and summarized how ncRNAs affect the regulators of m6A and by what means the expression of ncRNAs is altered *via* m6A in gastrointestinal cancers. We focused on the effect of the interaction of m6A and ncRNAs on the molecular mechanisms of malignant behavior in gastrointestinal cancers, revealing more possibilities of ncRNAs for diagnosis and treatment in term of epigenetic modification.

## 1 Introduction

Gastrointestinal (GI) cancers are responsible for over one-quarter of cancer-related morbidities and over one-third of mortalities from cancers worldwide (1), seriously affecting human health and wellbeing. GI cancers refer to a group of malignant tumors arising from the digestive tract, which mainly include esophageal cancer, gastric cancer, liver cancer, colorectal cancer, pancreatic cancer, and others (2–6). Smoking, infection, diet, and obesity are common risk factors to initiate the pathogenesis of the majority of GI cancers (7). Although the relevant treatment methods have been improved, the prognosis of patents is still unfavorable. Due to the growing incidence of these cancers in each year, there is a clear necessity of searching for early diagnostic markers and potential therapeutic targets (8, 9).

Recent research on epigenetic modifications has made numerous contributions to the understanding of the pathogenesis and biological characteristics of human diseases (10–13). N6-methyladenosine (m6A), first discovered in 1974 (14, 15), was not only observed as widely distributed in various eukaryotes, such as mammals, plants or yeast (16–19), but also located in bacteria, e.g., mycoplasma (20, 21). Studies on m6A have mainly focused on the consensus sequence RRACH (R = G or A and H = A, C, or U) that is near 3′ untranslated regions (3′-UTRs), 5′-UTR, and internal long exon (22). Methyltransferase and demethylase are responsible for the dynamic alteration of m6A modifications, respectively called “writers” (8, 9, 23) and “erasers” (24, 25). The binding protein (“readers”) (26, 27) can play a role in the occurrence of the methylation process mediated by “writers” and recruit downstream molecules to affect functional signaling. The role of these regulators in GI cancers have gradually attracted increasing attention. Methyltransferase-like 3 drives GLUT1 (Glucose transporter type 1) expression to accelerate glucose utilization and lactate accumulation in colorectal cancer cells (7). Furthermore, AlkBhomolog5 attenuates the stability of LY6/PLAUR domain-containing protein 1 (LYPD1) to constrain the aggressive ability of hepatocellular carcinoma through the elimination of m6A site (28). In addition, Ubiquitin-specific protease 14 (USP14) translation was reported to be upregulated by YTH N6-Methyladenosine RNA Binding Protein 1 (YTHDF1), resulting in carcinogenesis and unfavorable outcome for gastric cancer patients (29). The m6A modification site prevalently appears in mRNA (message RNA) to regulate its metabolism (30), such as biosynthesis, nuclear export and degradation. Increasing evidence has demonstrated that m6A can decorate various ncRNAs (non-coding RNAs) (31, 32), including microRNAs (miRNAs) (33–35), long noncoding RNAs (lncRNAs) (36–38) and circular RNAs (circRNAs) (39–41).

As transcriptomics studies have constantly shown great improvement, the function of non-coding RNAs (ncRNAs) have been gradually noticed and prompted further explorations (42, 43). Emerging evidence proves that several ncRNAs play indispensable roles in the initiation and progression of multiple diseases (44–47), and thus they have a potential value for diagnosis and prognosis. For instance, circACTN4 functions as a sponge to eliminate miR-424-5p to upregulate and recruit Yes-associated protein 1 (YAP1), which exerts a positive effect on inducing Frizzled-7 (FZD7) and in turn facilitates the development of intrahepatic cholangiocarcinoma (48). The phenotypic transdifferentiation of carotid artery smooth muscle cells (SMCs) to macrophage-like cells was reported to be regulated by the activation effect of KLF4 derived from lncRNA MIAT, and then developing into advanced atherosclerotic lesion (49). Hsa-miR-3178 activates the PI3K/Akt cascade to attenuate the sensitivity of PC patients to gemcitabine (50). When m6A modification occurs in the main ncRNAs, their expression and function are altered by specific proteins. The upregulated lncRNA LBX2−AS1 serves as a promoter of tumor by enhancing the proliferation and migration of colorectal cancer cells (51). Methyltransferase-like 3, the known “writers” of m6A, catalyzes and increases the m6A level in lncRNA LBX2−AS1 to enhance its expression by stabilizing LBX2−AS1 in colorectal cancer. Besides, a series of RNAs without translating proteins also affect the m6A modification level of downstream targets by interacting with critical regulators of m6A. Ubiquitin-conjugating enzyme E2T (UBE2T) has been considered as the pivotal target of lncRNA CASC11 to drive the malignant phenotype of hepatocellular carcinoma. On the one hand, lncRNA CASC11 could recruit ALKBH5 to reduce the m6A level in UBE2T mRNA, altering its stability. On the other hand, the interaction between YTHDF2 and UBE2T mRNA was prevented by highly expressed CASC11 (52).

In short, studies on the interplay of m6A and ncRNAs have gradually fallen into the public view, however, understanding the association between them on GI cancers still remains perplexed. Therefore, we summarized several special ncRNAs affecting the m6A level and vital effectors regulating ncRNAs expression in the same way as m6A. We concentrated on the interaction of m6A and ncRNAs in malignant behaviors of GI cancer and hoped that this review could provide promising targets for clinical treatment and promote the further development of epigenetics.

## 2 Regulators of m6A: “writers”, “erasers” and “readers”

Alternative effects derived from m6A are controlled by methyltransferases (“writers”), demethylases (“erasers”) and m6A-binding proteins (“readers”). The “writers” of m6A mainly consist of methyltransferase-like 3 (METTL3), METTL14, METTL16, METTL5, KIAA1429, Wilms’ tumor-1 associated protein (WTAP), zinc finger CCCH-type containing 13 (ZC3H13), zinc finger CCHC-type-containing 4 (ZCCHC4), and RNA-binding motif protein 15 (RBM15/15B) (23, 53–56). METTL3 was reported to predominantly catalyze the RNA of m6A deposition in 1997 (17), which has a capacity to bind to the methyl donor S-adenosyl-methionine (SAM) and achieve methyl transfer (57, 58). METTL14 is considered as an RNA-binding platform to collectively form a core methyltransferase complex with METTL3, catalyzing and generating m6A modification (54, 59). Meanwhile, WTAP functions as a vital adaptor protein to increase the stability of core methyltransferase complex (23, 60). As for RBM15/15B, it can bind to METTL3 and WTAP to recruit targets of the methyltransferase complex (53, 61). KIAA1429 was found to tend to locate the m6A site near the 3′-UTR and stop codon region (56). Different from the methyltransferase complex, certain reports demonstrated that METTL16, METTL5 and ZCCHC4 independently participate in the process of m6A decoration onto U6 snRNA, 28S rRNA and 18S rRNA (62–66). Abundance of methyltransferases lays the foundation for diversified regulation from m6A modification.

Regarding the research on “erasers”, only two proteins, Fat mass and obesity-associated protein (FTO) and AlkBhomolog5 (ALKBH5), were discovered to dynamically remove the m6A site (67, 68). As the firstly identified demethylase (67), FTO not only mediates m6A demethylation in many cell types but also can remove the m6Am (N6,20-O-dimethyladenosine) demethylase of mRNA (69, 70). ALKBH5 presents higher specificity than FTO and only catalyzes m6A modification to achieve demethylation (71). “Readers” are considered to recognize and bind to m6A sites on RNA, including YT521-B homology (YTH) domain-containing protein family, heterogeneous nuclear ribonucleoprotein (HNRNP) family, and K homology (KH) domain family (IGF2BP1/2/3, also named as IMP1/2/3) (72–74). YTHDC1, which belongs to the YTH domain-containing protein family, was found to be distributed in the nucleus. YTHDC2, YTHDF1, YTHDF2, and YTHDF3 are members of the YTH domain-containing protein family, located in cytoplasm. YTHDF1 was reported to enhance the efficiency of the initiation stage of RNA translation through interplay with initiation factors (75). Meanwhile, YTHDF2 acts in a pivotal role in RNA degradation *via* binding with mRNA. YTHDF3 also exerts a positive effect on translational efficiency by relying on synergy with YTHDF1 (76). Research on “readers” has been excavating more roles of m6A in hallmark pathways.

## 3 Interplay between m6A and noncoding RNAs

Noncoding RNAs (ncRNAs) lack the capacity of translating directly into protein but function as crucial transcripts to regulate gene expression (77, 78). On the basis of nucleotide length, ncRNAs are divided into multiple types, such as miRNAs, lncRNAs, circRNAs, snRNAs, rRNAs, and tRNAs, etc (79, 80). The m6A modification in these ncRNAs have been increasingly reported to have a close association with the pathological mechanisms of malignant tumors. One the one hand, several key regulators derived from m6A methylation influence ncRNAs to participate in the initiation and progression of cancers. On the other hand, ncRNAs could also mediate downstream targets to reversely affect the expression of “writers”, “readers” and “erasers” through m6A modification. Thus, further discussions on the interaction between m6A and ncRNAs provide guidance to comprehensively understand the mechanism of malignant behavior in GI cancers.

### 3.1 lncRNA to m6A

LncRNAs serves as a class of ncRNAs that are over 200 nucleotides in length, and are extensively considered to sponge miRNAs to exert a functional effect (81–83). Recent investigations revealed that lncRNAs could regulate the crucial protein of m6A modification to affect the accumulation of m6A methylation sites (84, 85). In colorectal cancer, lncRNA GAS5 was regarded to have the property of negatively modulating YTHDF3, leading to evidently weakened proliferative and invasive potential of cancer cells (86). Specifically, the negatively mediated effect was mainly achieved through the GAS5/YAP/YTHDF3 axis. YAP belongs to one of most essential components of Hippo signaling, and its phosphorylation could activate the Hippo pathway (87). A dysregulation of the Hippo cascade is required to drive tumorigenesis in colorectal cancer (88, 89). Various kinases are responsible for catalyzing different phosphorylated sites of YAP to regulate its distribution in the cytoplasm and nucleus (90, 91). GAS5 was found to induce ubiquitin-mediated degradation of YAP to inhibit Hippo/YAP signaling. Then, YAP could bind with promoter of YTHDF3 to actively improve transcriptional efficiency of YTHDF3. Overexpression of lncRNA GAS5 could decrease YTHDF3 expression by targeting YAP. But which YTDHF3 could regulate lncRNA GAS5 still remained uncharted.

Furthermore, Wang et al. revealed that lncRNA LINRIS mediated IGF2BP2 to take part in developmental process of colorectal cancer. The increased expression of LINRIS tended to emerge in colorectal cancer tissue compared to adjacent tissue and indicated dismal prognosis for patients. ILINRIS knockdown suppressed the proliferative rate of HCT116 and DLD-1 cells. Further investigation of the promotive effect from LINRIS in CRC showed that LINRIS interacts with IGF2BP2 to block its degradation and maintain stable expression. Numerous studies have reported that IGF2BP2 recognized mRNA with m6A modification to stabilize targeted mRNA, thus participating in the occurrence and development of various cancers (74, 92). When the anti-autophagy gene 5 (ATG5) was rapidly reduced, IGF2BP2 protein level was scarcely altered after knockdown of LINRIS. The result suggested that LINRIS affects IGF2BP2 degradation in the autophagic pathway. Hallmark molecules regulating glycolysis, like MYC, GLUT-1, PKM2 and LDHA, exhibited low levels as LINRIS was depleted. MYC mRNA has been widely acknowledged as the downstream factor of IGF2BP2. Overexpressed IGF2BP2 exerted the reversal effect on the downregulation of glycolysis activity *via* the LINRIS–IGF2BP2-MYC axis when LINRIS was silenced (93). Thus, it is also necessary to explore the LINRIS–IGF2BP2-MYC axis, which could yield significant improvement in the prognosis of colorectal cancer. The mediation to regulators generally is considered to indirectly effect and relative mechanism need to be deeper discussed.

### 3.2 m6A to lncRNA

**Figure 1:**
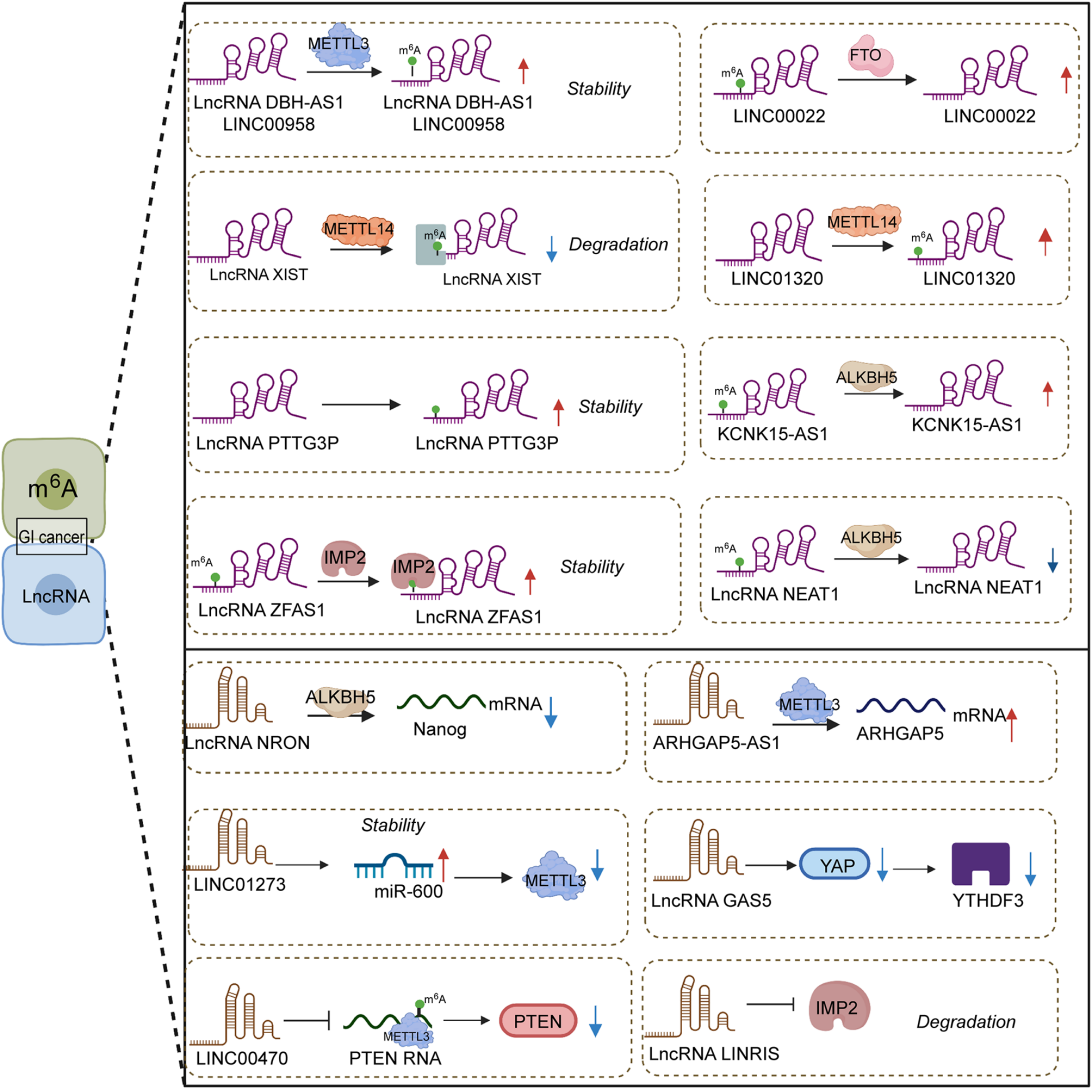
Interplay between lncRNAs and m6A in gastrointestinal cancers.

Abundant m6A sites were found in lncRNA, and their expression level was influenced through a specialized protein. Notably, differences in the methylation level of m6A also exerted contradictory effects on the expression of lncRNA owing to the regulation of respective targets. High m6A methylation could induce the downregulation of lncRNAs (**Figure 1**). For instance, the current study unraveled that lncRNA XIST expression was repressed by the binding of METTL14 and YTDHF2. Specifically, METTL14 elevated the m6A methylation level in XIST to suppress its expression through cooperating with YTDHF2. The corresponding mechanism of lncRNA XIST downregulation was confirmed, that is, YTDHF2 binds with the m6A complex from XIST to participate in the degradation process. In colorectal cancer, METTL14 was expressed lowly in cancer tissue compared with normal tissue, and METTL14 depletion was observed in patients with shorter survival times (94). The knockdown of METTL14 encouraged the biological property of proliferation and invasion by mediating lncRNA XIST.

In contrast, low methylation in lncRNA NEAT1 gave rise to a low-expression result. ALKBH5, the “eraser” of m6A, could catalyze the appearance of demethylation in lncRNA NEAT1 and its downregulation. In gastric cancer, NEAT1 might interact with EZH2 to reduce its expression, which was significantly related to the invasion and metastasis of tumors. Meanwhile, ALKBH5 silencing could recede the mentioned effect from NEAT1 (95). Similarly, LINC01320 upregulation might be induced by METTL14, and MEETL14 knockdown might trigger the loss of LINC01320; their positive correlation depends on the m6A modification degree. LINC01320 was seen as the activator of gastric cancer to affect its proliferative, invasive and migratory abilities *in vitro*. Malignant tumor behavior was triggered by LINC01320 overexpression *via* sponge miR-495-5p. It was acknowledged that RAB19 is downstream factor of miR-495-5p, and LINC01320 takes part in cell growth promotion through the LINC01320/miR-495-5p/RAB19 axis (96). The participation of METTL14 in the LINC01320/miR-495-5p/RAB19 axis deserves further investigation. Taken together, different effects from different m6A methylation levels in lncRNAs chiefly rely on the mediation of diverse targets.

### 3.3 miRNA to m6A

By definition, miRNAs are single-stranded ncRNAs with approximately 21-25 nucleotides, functioning as a RNA-induced silencing complex (RISC) to play a key role in the regulation of gene expression. As post-transcriptional regulators, miRNAs can reduce translation efficiency and impact mRNA degradation through the binding of 3′-UTR region from targeted mRNAs (96). One research reported that miR-4429 could inhibit METTL3 expression to play the role of suppressor in gastric cancer (97). The occurrence of m6A methylation mediated by METTL3 could stabilize SEC62 and exert a positive effect on SEC62 upregulation. Emerging evidence has demonstrated that m6A methylation depends on the promotion of binding between RBP and mRNA to increase mRNA stability (74, 98). METTL3 positively facilitated the binding between IGF2BP1 and SEC62 mRNA, stabilizing SEC62 mRNA in m6A-dependent manner. The lack of METTL3 caused the decline of m6A formation in SEC62. SEC62 was expressed highly in gastric cancer compared to normal tissue, and SEC62 knockdown elevated cell proliferation (99). Meanwhile, METTL3 upregulation was found to exert an analogous effect on participating in gastric cancer progression. MiRNAs not only affect “writers” but also “readers” and “erasers”.

MiR-6125 is considered as a promising biomarker for diagnosis and prognosis, and its decreased expression commonly implies an advanced tumor stage and poorer prognosis for colorectal cancer patients (100). *In vitro* and *in vivo* experiments testified that upregulation of miR-6125 prevents the proliferation phenomenon of colorectal cancer through the direct mediation of YTHDF2. MiR-6125 reduces the expression of cyclin D1 to generate the result of arrest at the G0/G1 phase of colorectal cancer cells, which depends on YTHDF2 mediating to take part in Wnt/β-catenin cascades. YTHDF2 serves as an oncogene to impact growth and malignant conversion in cancers (101–103). The ectopic expression of YTHDF2 in SW480 and RKO cells could enhance β-catenin accumulation. Aberrant accumulation from β-catenin gave rise to translocating from the cytoplasm to the nucleus, then modulating the cyclin D1 transcriptional process. The degradation of β-catenin was determined by the complex of Axin, GSK3β and APC. Notably, a deficiency of GSK3β attenuated the suppressive capacity of overexpressed miR-6125 in CRC cells. Therefore, miR-6125 mediated the degradation of β-catenin by affecting GSK3β (100). The role of miR-6125 and YTHDF2 in targeted treatment should be considered and the specific interaction between miR-6125 and YTHDF2 need more investigations and further discussion. MiR-96 could indirectly mediate FTO to affect the aggressive and malignant capacity of colorectal cancer. It was reported that FTO expression was downregulated by AMPKα2. AMPKα2 (104) has been considered as the target of miR-96 to impair proliferation, migration, and invasion in colorectal cancer. FTO could decrease the methylation of MYC mRNA to accelerate MYC accumulation. miR-96 indirectly mediated AMPKα2 to achieve MYC upregulation to enhance the aggressive ability of colorectal cancer, which can be a promising novel target to prolong survival time of patients (105).

### 3.4 m6A to miRNA

**Figure 2:**
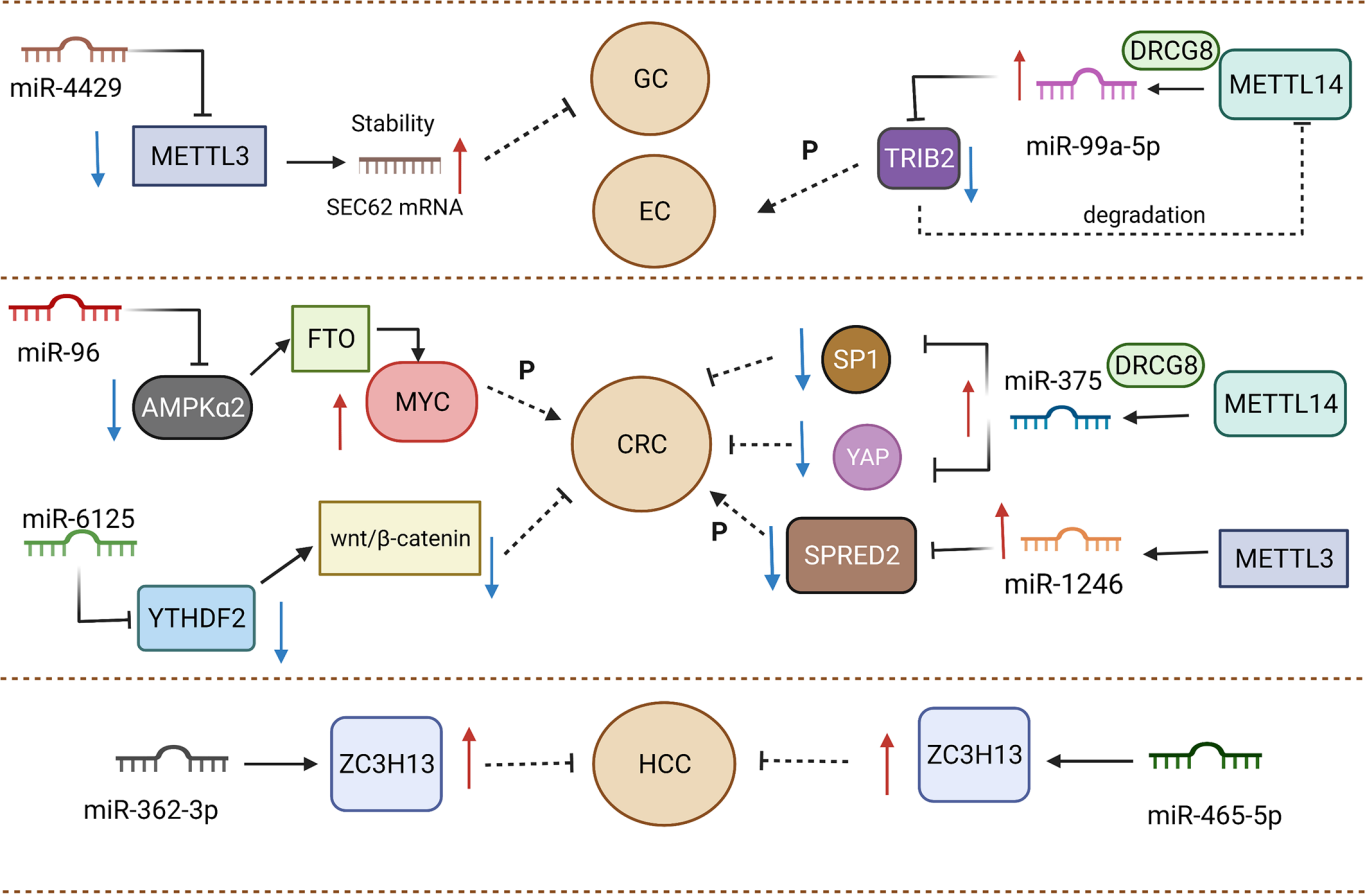
Various miRNAs and m6A regulators play promoting or inhibiting roles in gastrointestinal cancers.

The formation of miRNA genes is commonly considered to initially undergo transcription mediated by RNA polymerase II promoters and maturation by further processing (106). Microprocessors, like the DiGeorge syndrome chromosomal region 8 (DGCR8), take part in processing into precursor miRNAs (pre-miRNAs) and subsequently Dicer carved pre-miRNAs to form mature single-stranded miRNAs (107). METTL14 was reported to regulate miR-375 processing *via* DGCR8. The number of unprocessed miR-375 increased after the silencing of METTL14. As the writer of m6A, METTL14 served as an antitumor factor of colorectal cancer to affect patient prognosis. The depletion of METTL14 might cause improvement of proliferative, invasive and migrative ability in colorectal cancer cells. The inhibiting effect from METTL14 was found to have close association with miR-375. Overexpression of METTL14 dramatically upregulated miR-375 as well as decreased YAP1 and SP1 expression in HCT116 and HCT8 cells. Therefore, METTL14 could participate in miR-375/YAP1 and miR-375/SP1 to mediate the inhibition of colorectal cancer progression *via* the manner of m6A modification (108). In addition, Peng et al. demonstrated that silencing METTL3 reduces the expression of mature miR-1246 and increases pri-miR-146 accumulation. When METTL3 was overexpressed, the above result could be reversed, which implied that METTL3 might facilitate the transformation of pri-miR-146 into miR-1246 (109). In colorectal cancer tissue, a clearly elevated m6A modification level was responsible for METTL3. To sum up, effects from regulators of m6A mainly focus on processing program in biogenesis aspect of miRNAs (**Figure 2**).

### 3.5 circRNA to m6A

CircRNAs, firstly described in 1976 (110), are regarded to originate from pre-mRNA back-splicing (111) and have a special ring structure (111, 112), the formation of which principally depends on 3′,5′-phosphodiester bond connecting at the 3′-end and 5′-end or an upstream exon (113, 114). As ncRNAs with regulatory potency (115, 116), circRNAs with differential expression have been observed in numerous diseases, and they have become known as significant modulators of pathogenesis (117–120). Wang et al. (120) reported that enhancing circRNA-KIAA1429 activity resulted in accelerating migration, invasion and EMT progression of hepatocellular carcinoma cells. The carcinogenic role of cirRNA-KIAA1429 upregulating the downstream target Zeb1 is yet to be established. The expression of Zeb1 was confirmed to be mediated by YTHDF3 through m6A chemical modification. YTHDF3, acting as a “reader” of m6A, stabilized Zeb1 mRNA and prolonged its lifespan. When YTHDF3 expression was decreased, the effect of overexpressed cirRNA-KIAA1429 also was attenuated *via* depletion of Zeb1, suggesting that cirRNA-KIAA1429 cooperated with YTHDF3 to affect tumor development.

Emerging evidences have revealed that circRNAs function as a sponge to absorb miRNA, implicating their involvement in the biological process of multiple cancers (121). In fact, several circRNAs establish correlations with miRNA to indirectly mediate the expression of m6A regulators. High abundance of circPTK2 was observed in advanced stages of colorectal cancer patients, and miR-136-5p expression was decreased to induce chemoresistance. YTHDF1 was seen as the effective target of miR-136-5p. At the same time, enforced expression of miR-136-5p could adversely modulate YTHDF1 to suppress the proliferation and improve the sensitivity of colorectal cancer to chemotherapy. CircPTK2 indirectly upregulated YTHDF1 *via* the elimination of miR-136-5p (122). Additionally, Chi et al. testified that the role of circMAP2K4/hsa-miR-139-5p/YTHDF1 axis in the growth of hepatocellular carcinoma also verified the above-mentioned hypothesis. The inhibition of proliferative ability in hepatocellular carcinoma by hsa-miR-139-5p was partly reversed by highly expressed cirMAP2K4. The promotion of upregulated cirMAP2K4 sponged hsa-miR-139-5p to increase YTHDF1 activity, resulting in stimulating development and progression of hepatocellular carcinoma (123). To sum up, circRNAs are involved in m6A regulation with the aid of miRNA and “readers”, which enriches the existing studies on the molecular mechanism of circRNAs function at the post-transcriptional level.

### 3.6 m6A to circRNA

**Figure 3:**
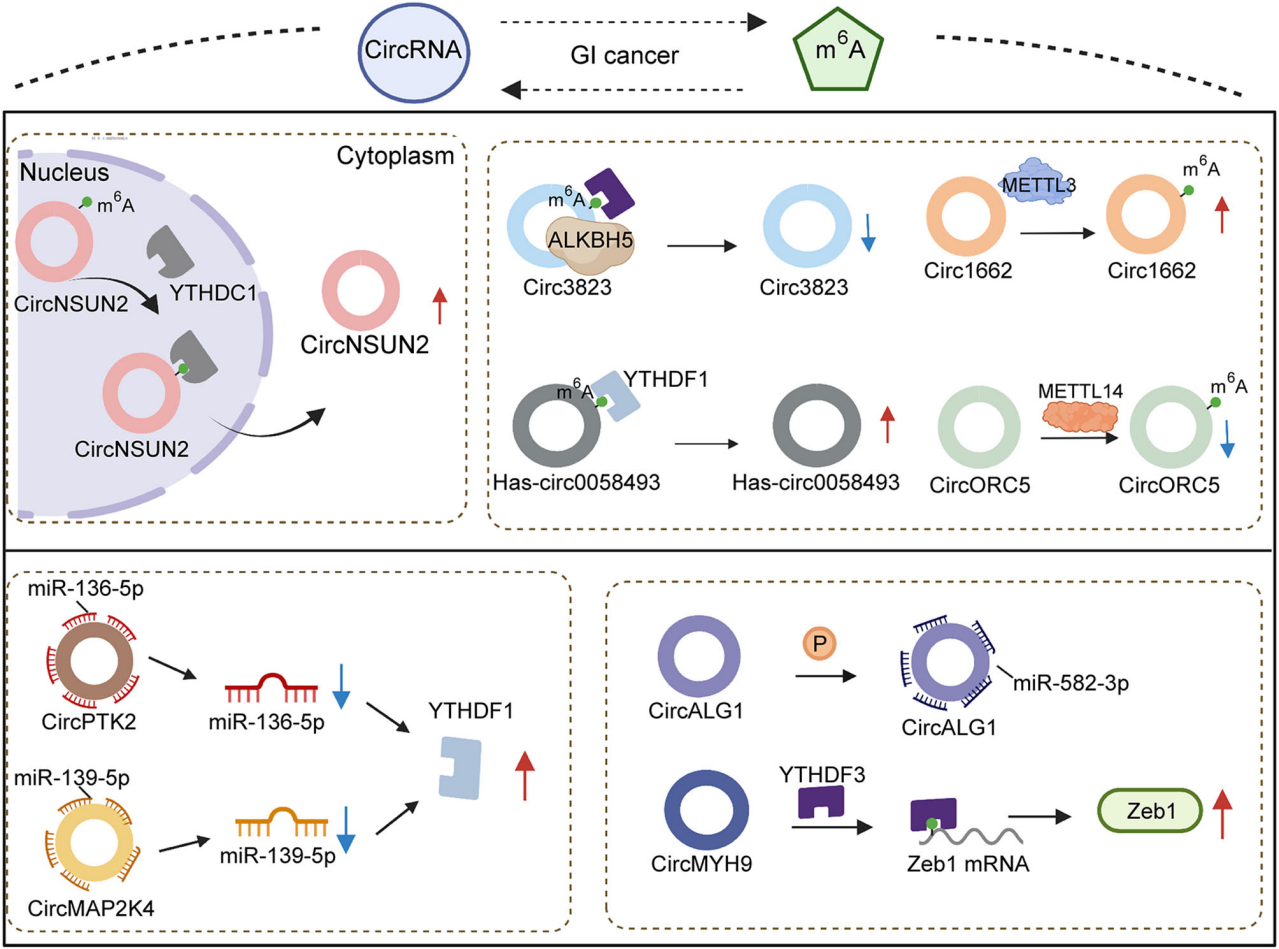
Association of circRNAs and m6A regulators in gastrointestinal cancers.

Canonical splicing is widely regarded as a mechanism of circRNA formation, depending on the splice signal sites and spliceosomes (124). The participation of reverse complementary sequences in intron flanking sequences was regarded to play a critical role in the process of circRNA biogenesis (125) (**Figure 3**). A recent study demonstrated that inverted repeated Alu pairs located in flanking introns exerted the effect of fostering extrusion of flanking introns and splicing process (126). Emerging evidence testified that a series of RNA bind proteins (RBPs) bind with flanking introns of circRNA to involve in the alteration of circRNA expression, which contain draw introns (127, 128) and modify the stability of Alu pairs (129, 130) to promote or inhibit circularization. METTL3 was confirmed to form m6A in flanking reverse complementary sequences of circ1662, remarkably elevating the expression of circ1662 in colorectal cancer. Overexpressed circ1662 could induce the invasion, migration and EMT of hepatocellular carcinoma cells *in vitro*. Increasing the invasive ability of a tumor is considered to easily trigger cancer metastasis. Circ1662 was found to improve the efficiency of nuclear transport of YAP1 to downregulate SMAD3, causing the promotion of tumor aggressiveness. Thus, METTL3 was proved to have an impact on circ1662 formation to drive colorectal cancer metastasis (131). An existing report revealed that knockdown of METTL14 contributed to the reduction of m6A sites in circORC5 and upregulate circORC5 (3). Meanwhile, whether the regulation of METTL14 to circORC5 has a dependency on flanking introns of circRNA remains unclear. In gastric cancer the capabilities of tumorigenesis and invasion from highly expressed METTL14 could be impaired by circORC5 deficiency. CircORC5 functions as a sponge to eliminate miR-30c-2-3p expression to participate in the malignant progression of gastric cancer. Numerous reports have indicated that miR-30c-2-3p downregulation has a negative effect on cell proliferative ability and the normal cycle of pancreatic cancer (132) and breast cancer (133). Collectively, METTL14 alters m6A density of circORC5 to negatively modulate its expression, followed by mediating the miR-30c-2-3p/AKT1S1 axis to induce the occurrence of malignant behavior in gastric cancer. Different from miRNA, regulators of m6A have been reported to affect nuclear export as well as biogenesis.

CircRNA has been commonly considered to occur in the cytoplasm (134), thus circRNAs located in the nucleus depend on particular proteins to accomplish nuclear export. For instance, it was reported that YTHDC1 could control circNSUN2 translocation (135). YTHDC1 bonds with the GAACU m6A motif of circNSUN2 to stimulate its export from nucleus to cytoplasm. When YTHDC1 expression was decreased, circNSUN2 expression in the nucleus subsequently showed abundant accumulation. Upregulated circNSUN2 was frequently observed in the serum of colorectal cancer patients with liver metastasis and predicted shorter survival times. Notably, the promotion of tumor aggression from circNSUN2 mediated IGF2BP2 and downstream targets to attain, instead of affect, NSUN2 expression. Regulation derived from m6A to circRNA is a better and more comprehensive cognition of circRNA metabolism and function.

## 4 Association of m6 with non-coding RNAs in various GI cancers

**Figure 4:**
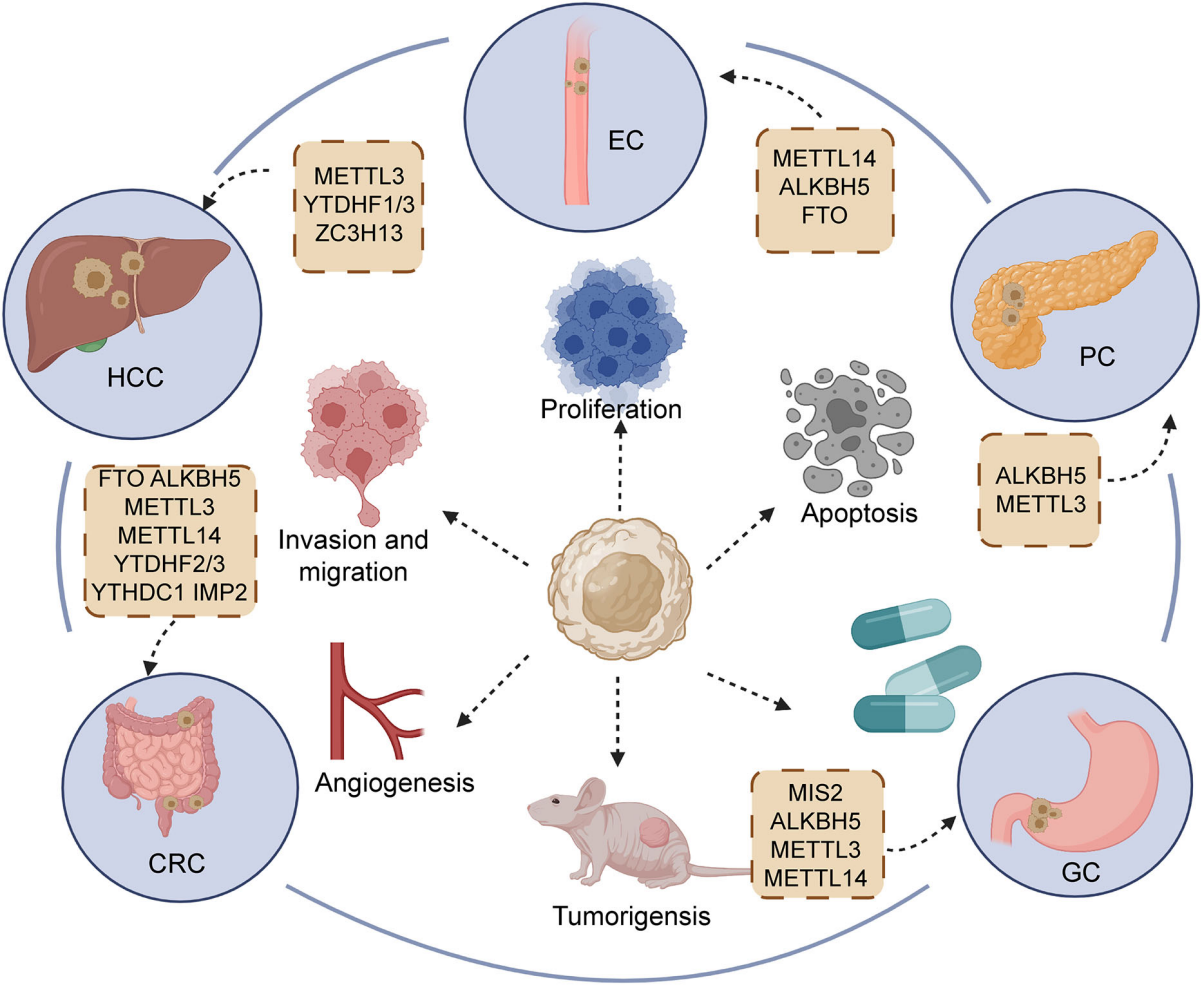
Regulators of m6A participating in the initiation and development of main gastrointestinal cancers, including esophageal cancer (EC), hepatocellular carcinoma (HCC), gastric cancer (GC), pancreatic cancers (PC), and colorectal cancer (CRC).

In GI cancers, the m6A modification level is related to clinical characteristics and has a contradictory impact on the prognosis of tumors. Here, according to a variety of GI cancers, we sorted out ncRNAs with m6A modification and further concluded the carcinogenic or anti-cancer effects of different ncRNAs (**Table 1**; **Figure 4**).

**Table 1:** Mechanism of interaction between main ncRNAs and m6A in gastrointestinal cancers.

ncRNAs	Biological function	Molecules	Interaction mechanism	Ref.
LINC00022	Promoting cell proliferation in vitro and tumorigenesis in vivo	FTO	Enhancing LINC00022 expression via reduction of m6A	(2)
MiR-194-2	Promoting cell proliferation, migration in vitro and tumorigenesis in vivo	ALKBH5	Downregulating miR-194-2 by preventing maturation of pri-miR-194-2	(136)
MiR-99a-5p	Promoting cancer stem cell persistence and radio-resistance	METTL14	Promoting miR-99a-5p expression by maturing pri-miR-99a-5p	(137)
LINC00958	Promoting cell proliferation, invasion, migration, and lipogenesis	METTL3	Promoting LINC00958 expression by stabilizing LINC00958	(138)
LINC01273	Inhibiting cell chemoresistance	METTL3	Downregulating METTL3 expression via LINC01273	(138)
LncRNA DUXAP8	Inducing sorafenib resistance	METTL3	Promoting DUXAP8 expression via METTL3	(139)
MiR-582-3p	Inhibiting tumorigenesis in vivo	/	Promoting the binding of miR-582-3p and YAP by high m6A modification	(140)
MiR-362-3p/miR-425-5p	Reducing infiltration of tumor immune cell	ZC3H13	Downregulating ZC3H13 via MiR-362-3p and miR-425-5p	(141)
Has-circ-0058493	Promoting cell proliferation and invasion in vitro and growth in vivo	METTL3, YTHDF1	Enhancing has-circ-0058493 expression	(142)
CirMAP2K4	promoting cell proliferation	YTHDF1	Promoting YTHDF1 expression via cirMAP2K4	(123)
LncRNA ARHGAP5-AS1	Increasing cell proliferation and chemoresistance and inhibiting cell apoptosis	METTL3	Recruiting METTL3 to upregulate ARHGAP5 via ARHGAP5-AS1	(143)
LINC00470	Promoting cell proliferation, invasion, and migration	METTL3	Preventing the binding of METTL3 and PTEN via LINC00470	(144)
LncRNA NRON	Promoting growth in vitro and vivo	ALKBH5	Recruiting ALKBH5 to stabilize Nanog mRNA via lncRNA NRON	(144)
LncRNA NEAT1	Inhibiting invasion in vitro	ALKBH5	Inhibiting NEAT1 expression via downregulation of m6A level	(95)
LINC01320	Promoting cell proliferation migration, and invasion	METTL14	Enhancing LINC01320 expression	(96)
LINC00942	Promoting chemoresistance in vitro and vivo	MSI2(IGF2BP2)	Promoting MSI2 expression via LINC00942	(145)
MiR-4429	Preventing cell proliferation	METTL3	Inhibiting METTL3 expression via MiR-4429	(97)
LINC00857	Inducing proliferation and impeding apoptosis	/	Stabilizing LINC00857 expression by high m6A level	(146)
LncRNA KCNK15-AS1	Inhibiting proliferation and promoting apoptosis	ALKBH5	Upregulating KCNK15-AS1	(147)
LncRNA DBH-AS1	Inhibiting growth and gemcitabine resistance	METTL3	Promoting DBH-AS1 expression by stabilizing DBH-AS1	(148)
LncRNA ZFAS1	Promoting cell glycolysis	IMP2	Enhancing ZFAS1 expression by increasing ZFAS1 stability	(149)
LncRNA PTTG3P	Promoting cell glycolysis	METTL3, IGF2BP2	Promoting PTTG3P expression via METTL3 and IGF2BP2	(150)
LncRNA GAS5	Inhibiting cell proliferation and invasion invitro and tumorigenesis in vivo	YTHDF3	Inhibiting YTHDF3 expression via lncRNA GAS5	(86)
LncRNA LINRIS	Inhibiting cell glycolysis	IGF2BP2	Enhancing stability of IGF2BP2 via lncRNA LINRIS	(93)
LncRNA XIST	Promoting proliferation and invasion	METTL14, YTDHF2	Reducing lncRNA XIST expression by mediating degradation	(151)
MiR-6125	Preventing cell proliferation	YTDHF2	Inhibited YTDHF2 expression via miR-6125	(100)
MiR-96	Promoting cell proliferation, migration and invasion and inhibiting cell apoptosis	FTO	Promoted FTO expression via miR-96	(105)
MiR-375	Inhibiting growth and metastasis in vitro and vivo	METTL14	Upregulating miR-375 by promoting maturation of pri-miR-375	(108)
MiR-1246	Promoting invasion and migration in vitro and metastasis	METTL3	Enhancing miR-1246 expression by promoting maturation of pri-miR-1246	(109)
CircALG1	Promoting metastasis in vitro and vivo	/	Elevating circALG1 expression and promoting the binding of circALG1 and miR-342-5p via high m6A level	(152)
CircPTK2	Promoting cell proliferation, migration, invasion and chemoresistance	YTDHF1	Promoting YTDHF1 expression via CircPTK2	(122)
Circ1662	Promoting cell invasion, migration and EMT in vitro	METTL3	Upregulating circ1662	(131)
CircORC5	Inhibiting cell proliferation and invasion in vivo and tumorigenesis in vivo	METTL14	Reducing circORC5 expression	(3)
CircNSUN2	Promoting cell invasion and migration	YTHDC1	Upregulating circNSUN2 expression by enhancing circNSUN2 export	(135)
Circ3823	Promoting cell proliferation, invasion, and angiogenesis	YTHDF3, ALKBH5	Inhibiting circ3823 expression via promoting degradation	(153)

### 4.1 Esophageal cancer

In spite of advancements in multidisciplinary antitumor treatment for esophageal cancer (EC), the 5-year survival rate of patients still shows a negative trend, owing to uncharted molecular mechanisms and uncontrolled recurrence (154, 155). Accumulating evidence demonstrates that m6A modification plays a critical role in ncRNA function to affect the biological behavior of EC. Cui et al. (2) uncovered the elevated expression of LINC00022 and shortened survival time in patients with advanced-stage esophageal squamous cell carcinoma (ESCC). FTO overexpression obviously reduced the m6A methylation level in ESCC, and LINC00022 expression exhibited accumulation due to the high expression of FTO. The alteration of cell cycle plays a pivotal role in the proliferative progression of multiple malignancies (2, 156). LINC00022 knockdown was shown to affect the distribution of cell cycle, mainly manifesting in the increase of cells in G0/G1 and reduction of cells in the G2/M phase. In addition, several inhibitors of CDK, including p16, p21 and p53, remained upregulated in KYSE150 and TE1 cell owing to the silencing of LINC00022. Meanwhile, upregulated FTO could positively mediate LINC00022 to reverse the above-mentioned phenomenon. Additionally, the expression of YTHDF2 was also confirmed to influence the FTO/LINC00022 axis. YTHDF2 directly bind with LINC00022 transcript to affect stability and promote degradation. “Eraser” ALKBH5 is fame antitumor factor in hepatocellular and pancreatic cancer (28, 157). In EC, ALKBH5 serves as a positive prognostic factor to predict the survival time of patients (136). DGCR8 is recruited by the demethylase activity of ALKBH5, participating in the processing of pri-miR-194-2. Overexpression of ALKBH5 could prevent DGCR8 from binding with pri-miR-194-2. ALKBH5 repressed the downregulation of RAI1 caused by miR-194-2, contributing to the release of RAI1 and its inhibition of malignancy. RAI1 was confirmed to promote tumor development through participation in the Hippo pathway, which could bind with the DNA region of Hippo signaling to upregulate the expression pathway and block the nuclear translocation of YAP/TAZ (158–160). Thus, the ALKBH5/DGCR8/miR-194-2/Hippo axis to EC targeted treatment opens up a novel orientation. In EC, regulators of m6A modification are able to affect biogenesis and degradation of ncRNAs, which provide a novel orientation to clinical treatment of EC.

### 4.2 Hepatocellular carcinoma

Hepatocellular carcinoma (HCC), the most common type of primary liver cancer, greatly threatens human health greatly by its striking morbidity and mortality (161). Compared with adjacent normal tissue, increased m6A modification was found in the YAP 3′UTR region of HCC tissue. Notably, m6A methylation plays an indispensable role in inducing the interaction of miR-582-3p with YAP. The overexpression of circ-104075 could elevate the expression of YAP in mRNA and protein level by absorbing miR-582-3p, contributing to the promotion of tumorigenesis in HCC (140). Analogously, METTL3 catalyzes the formation of m6A in has-circ-0058493, followed by causing the binding of YTHDF1 with has-circ-0058493. YTHDF1 dramatically enhanced the exporting efficiency of has-circ-0058493 from the nucleus to the cytoplasm depending on the m6A modification, which promoted the growth and malignant progression of HCC (142). KIAA1429, composed of METTL3, METTL14 and WTAP, is responsible for RNA m6A methylation, mainly including the regions of 3′ UTR and near the stop codon (56, 162). Highly expressed KIAA1429 was found to closely correlate with poorer prognosis for HCC patients. KIAA1429 mediates GATA3 to exert the effect of fostering development and metastasis of HCC both *in vivo* and *in vitro*. GATA3 functions as the famous transcript factor to stimulate the activation of multiple tumor suppressors (72, 163). With the help of LncRNA GATA3-AS, KIAA1429 gives rise to the occurrence of m6A methylation in the GATA3 precursor mRNA (pre-mRNA), which causes the interruption of binding between GATA3 pre-mRNA and HuR to enhance the degradation of GATA3 pre-mRNA, subsequently downregulating GATA3 (164). In contrast, KIAA1429 deficiency could recover the interaction of HuR and GATA3 pre-mRNA, leading to maintained stability of GATA3 pre-mRNA to block the malignant behavior of HCC. In short, we can notice that “writers” and “readers” commonly have an impact on phenotype of tumor in most cases.

“Writers” of the m6A modification are also affected by ncRNAs to participate in the complex mechanism of HCC. For instance, the ZC3H13 “writer” is negatively mediated by miR-362-3p/miR-425-5p mimics. Meanwhile, inhibitors of miR-362-3p/miR-425-5p could increase the expression of ZC3H13. In short, ZC3H13 was confirmed as a target of miR-362-3p/miR-425-5p. ZC3H13 might accelerate immune cell infiltration in HCC to affect the prognosis of patients. Downregulated ZC3H13 could predict adverse outcomes in HCC (141). Nevertheless, more research is required to explore which biological behaviors of tumor are affected by ZC3H13 and how miR-362-3p/miR-425-5p mediate ZC3H13. These findings for HCC focused on the fact that “writers” and “readers” are affected by ncRNAs or their mediating effects on ncRNAs. More research is needed to explore the regulators of “erasers” and ncRNAs in hepatocellular carcinoma.

### 4.3 Gastric cancer

Gastric cancer (GC) is considered as the fifth most frequent category of tumors, and its mortality in cancers ranks fourth globally (1, 165). Various researches have been performed on m6A and ncRNAs to probe the mechanism of malignant phenotype in GC. Wang et al. demonstrated that lncRNA NRON facilitated the proliferative capacity *in vitro* and *in vivo via* the modulation of ALKBH5 and Nanog (166). It is widely acknowledged that ALKBH5 strictly regulates the m6A methylation of Nanog (167, 168). LncRNA NRON could recruit ALKBH5 to enhance the stability and prohibit the degradation of Nanog mRNA. In GC, LINC00470 was found to be highly expressed and associated with dismal prognosis. This is because LINC00470 mediates the degradation of PTEN mRNA to decrease the expression of PTEN. Specifically, LINC00470 exerted an impact on the process of binding between METTL3 and PTEN to prevent the occurrence m6A methylation in PTEN (144). PTEN is implicated in the homeostasis of PI3K/AKT cascade to serve as a protective factor of tumors. The reduction of PTEN might induce malignant behavior in human cancers, such as proliferation, angiogenesis and migration (169). Interestingly, YTHDF2 also participates in process of shortening the half-life of PTEN induced by LINC00470. The effect of downregulating PTEN by overexpressing LINC00470 could be restored after silencing YTHDF2 (144). Furthermore, ncRNAs could mediate m6A regulation to influence the formation of chemoresistance. The upregulation of lncRNA ARHGAP5-AS1 was reported to stimulate the expression of ARHGAP5, resulting in attenuating the drug-sensitivity of gastric cells to cisplatin (DDP), ADM and 5-FU. Mechanistically, ARHGAP5-AS1 recruits METTL3 to enhance m6A modification, effectively promoting the transcription of ARHGAP5 and increasing the stability of ARHGAP5 mRNA in the cytoplasm (143). ARHGAP5 depletion reversed the chemotherapy resistance of gastric cells by inducing the activation of autophagy and increasing intracellular drug concentration.

### 4.4 Pancreatic cancer

As one of top four life-threatening malignant tumors worldwide (170, 171), the 5-year survival rate of pancreatic cancer (PC) of patients is low at approximately 5% (172). The options for PC clinical treatment are mainly surgical resection and chemotherapy approaches (173, 174). Owing to the late diagnosis, a number of patients are unable to attain a satisfactory outcome (172). Thus, new therapeutic targets and early diagnosis are necessary. LINC00857 was discovered to be highly expressed in adverse prognosis of HCC patients. Overexpression LINC00857 could induce proliferation and impede the apoptosis of PC cells by directly absorbing miR-150-5p. It was reported that high m6A methylation rate appeared in LINC00857 and increased its stability in HCC tissue, as compared with cancer adjacent tissue (146). The upregulation of potassium two pore domain channel subfamily K member 15 and WISP2 antisense RNA 1 (KCNK15-AS1) could hamper the growth and stimulate the apoptosis of PC cells. Consistently, the overexpression of KCNK15-AS1 improved epithelial marker (E-cadherin) expression and adversely mediated mesenchymal marker (N-cadherin) expression, remarkably blocking the EMT process. Due to the lack of ALKBH5, the m6A in KCNK15-AS1 presented an elevated level but KCNK15-AS1 expression manifested a decreased level. Collectively, ALKBH5 could mediate m6A methylation to positively upregulate KCNK15-AS1 (147). However, the detailed mechanism of low m6A methylation improving KCNK15-AS1 expression remains unknown. Investigations on m6A and ncRNAs in pancreatic cancer are scarce, while the potential of regulators and ncRNAs should not be ignored.

### 4.5 Colorectal cancer

Colorectal cancer (CRC), serving as the predominant cause of cancer-related deaths worldwide (175, 176), easily presents parenteral metastasis and thus brings unsatisfactory prognosis (177). Lin et al. (152) observed that circALG1 activity was upregulated in the tumor tissue and peripheral blood of CRC patients. MiR-342-5p could exert a negative effect on regulating placental growth factor (PGF) expression *via* binding with the 3’UTR region of PGE. CircALG1 facilitated the expression of PGF by sponging miR-342-5p. M6A modification not only elevated circALG1 expression but also reinforced the binding between circALG1 and miR-342-5p. However, the specific mechanism of m6A methylation affecting circALG1 expression remains unknown.

IMP2, a m6A reader expressed in CRC tissues, was discovered to have a close association with lncRNA ZFAS1. The dysfunction of IMP2 contributed to altering ZFAS1 expression. When IMP2 was expressed at a low level, ZFAS1 half-life became shorter in HCT116 and SW620 CRC cells. It was confirmed that IMP2 could directly bind with ZAFAS1 in the KH3-4 domain to activate its expression by intensifying ZAFS1 stability. The m6A modification was regarded to connect IMP2 and ZAFS1. High expression of ZAFS1 could improve the ATPase activity of OLA1 to positively further influence the formation of lactic acid and release of raw material to ATP synthesis, triggering glycolytic signaling to stimulate malignant progression in CRC (149).

Furthermore, METTL3 was validated to enhance m6A modification in lncRNA pituitary tumor-transforming 3 pseudogene (PTTG3P) to promote its expression. The stability of PTTG3P could be strengthened by METTL3 in a m6A-dependent manner. When IGF2BP2 was depleted, its upregulating effect on PTTG3P was also weakened to some extent. METTL3 positively mediated PTTG3P expression with the collaborative participation of IGF2BP2 (150). Overexpression of PTTG3P accelerated glycolysis of CRC. It is well-known that glycolysis occurs faster in malignancy than normal tissue (178–180). The accumulation of tumor lactate has been testified to cause distant metastasis and tumor return (181, 182). Collectively, the activity of METTL3 and IGF2BP2 could have an impact on the malignant phenotype of CRC *via* PTTG3P. In CRC, more research demonstrated that “writers” and “readers” take part in complex mechanism of initiation and malignancy. These regulators of m6A should be important therapeutic targets regarding the control of ncRNA function.

## 5 Clinical implications of m6A modification in ncRNAs

### 5.1 Potential biomarkers in GI cancers

Early diagnostic markers not only identify the type of cancers faster but lay the foundations for radical surgical treatment. Effective prognosis markers can reflect the probability of metastasis and recurrence, better judging the benefits from surgery and monitoring the status of patients post-surgery. Accumulating knowledge has provided new insights into m6A modification in ncRNAs (183, 184), which hopefully offer novel biomarkers for prompt diagnosis and prognosis in gastrointestinal tumors. Liquid biopsy is considered as a non-invasive and low-cost detection method, which could diagnose tumors by analyzing the appropriate biomarkers existing in blood, urine, ascites, cerebrospinal fluid, and so on (184, 185). In the serum of CRC patients, circ3823 (153) was observed to present a high level with remarkable specificity and sensitivity. This finding implies that the accurate and timely diagnosis of colorectal cancer benefits from detecting circ3823 from liquid biopsy. Guo et al. (153) reported that elevated expression of circ3823 had a close association with tumor stage and lymph node metastasis, because circ3823 weakens miR-30c-5p expression to boost tumor proliferation and invasion both *in vitro* and *in vivo*. Therefore, circ3823 could also serve as the satisfactory prognosis marker in CRC. Altered circ3823 expression in CRC was affected by YTHDF3 and ALKBH5 mediated by m6A modification, which might be related to the induction of circ3823 degradation, while this presumption needs more experiments to be verified. Analogously, LINC00460 expression also plays an indispensable role in prognosis prediction for patients with CRC (151). Upregulated LINC00460 tended to occur in advanced stages of tumor, because LINC00460 could heighten the proliferative and invasive ability of CRC cells. Tumor growth and lung metastasis would be arrested after silencing of LINC00460 (151). HMGA1 was further proved to be an important downstream regulator of LINC00460. Specifically, LINC00460 interacted with IGF2BP2 and DHX9 to recognize the m6A site mediated by METTL3 in HMGA1, contributing to stabilizing HMGA1 mRNA at the post-transcriptional level (151). With the assistance of m6A modification, the expression of numerous ncRNAs is altered to reflect their differential distribution in malignant and normal diseases. Besides, differences in the expression of special ncRNAs were testified to be significantly correlated to clinical features of cancer, which enlarged the horizons of knowledge on the occurrence and development of tumors.

### 5.2 Therapeutic targets in GI cancers

The continuous development of nanotherapeutics has brought great hope for the precise and effective treatment of malignant tumors (186–188). For example, taking polymeric nanoparticle (NP) to deliver siRNA might reduce its degradation and maintain its continuous release (189). It was reported that PLGA-based NPs incorporating FAK siRNA obviously improved the sensitivity of ovarian cancer patients to chemotherapy (190). Overexpressed LINC00958 enhances the proliferative capacity of Hep3B and HepG2 cells by mediating miR-3619-5p (138). PLGA-PEG (si-LINC00958) NP was demonstrated to rapidly block tumor growth and development in HCC. Moreover, ETTL3 expression was revealed to have a compact correlation with LINC00958 expression. For instance, LINC00958 expression would present a decline as METTL3 was silenced, which was related to the stabilization of LINC00958 transcripts from m6A induced by METTL3 (138). Thus, the exploration and utilization of interactions between m6A modification and LIC000958 might shed a new light on NP and enrich therapeutic approaches in HCC (138).

Nanotherapeutics has prosper development prospect, but conventional chemotherapy is still the cornerstone of treatment of unresectable HCC. For instance, sorafenib, a tyrosine kinase inhibitor, has been become one of preferred drugs for chemotherapy in patients with an unfavorable stage of tumor (191–193). However, the sorafenib resistance of HCC renders therapeutic effect descending. Current studies confirmed that lncRNA DUXAP8 (139) and LINC01273 (194) are linked to the occurrence of sorafenib resistance. It was discovered that LINC01273 knockdown can impair cell viability and DNA synthesis to increase the sensitivity of tumor to sorafenib (194). At the same time, the above effect could be attenuated due to the downregulation of METTL3. LINC01273 negatively regulated METTL3 expression by stabilizing miR-600. Conversely, METTL3 overexpression also had a suppressing impact on LINC01273 expression. The LINC01273 level was controlled by YTDHF2, and YTDHF2 depletion might enrich LINC01273 to decrease sorafenib sensitivity in a m6A-dependent manner (194). Likewise, DUXAP8 competitively bonded with miR-584-5p to trigger MAPK/ERK signaling, resulting in the chemoresistance of HCC against sorafenib (139). Different from LINC01273, METTL3 exerted an accelerative effect on DUXAP8 expression to affect sorafenib resistance through mediation by m6A (139).

A group of lncRNAs also participate in other complex mechanisms of chemotherapy-drug resistance, including gemcitabine (148) and cisplatin (DDP) (145). It was reported that gemcitabine serves as the first-line drug to treat patients of pancreatic cancer in the malignant stage (195, 196). However, the fact is that the phenomenon of gemcitabine chemoresistance in patients has been prevalent in clinical treatment practice. Decreasing expression of lncRNA DBH-AS1 was observed in gemcitabine-resistant pancreatic cancer tissues and cells (148). Mechanically, it was reported that DBH-AS1 sequestered miR-3163 to induce USP44 upregulation, leading to the enhancement of gemcitabine sensitivity and benefiting prognosis of patients. The study demonstrated that variation of lncRNA DBH-AS1 expression was affected by m6A modification level in the degreed (148). METTL3 catalyzed m6A modification in DBH-AS1 to stabilize DBH-AS1. DBH-AS1 expression would present downregulation when METTL3 was knockdown (148). In gastric cancer, the use of cisplatin alone or combined are predominant chemotherapeutic methods for patients with a lack of surgery opportunity (197, 198). Unlike DBH-AS1, overexpression of LINC00942 presented in gastric cancer cells of DDP resistance. LINC00942 was able to inhibit apoptosis and stimulate stemness, initiating DDP resistance. Specifically, because LINC00942 bind with MSI2 (Musashi2) to hamper degradation from SCFβ‐TRCP E3 ubiquitin ligase, MIS2 stability gained increasing (145). *c‐Myc* is a well‐known target of chemotherapy resistance in multiple cancers (199). MSI2 stabilized *c‐Myc* RNA depended on m6A modification. What is noteworthy is that the binding between c‐Myc and MISI was affect by expression of METTL3‐METTL14–WTAP complex (145). LINC00942/MIS2/c‐Myc axis will become the potential targets to elevate DDP treatment efficiency for patients.

The phenomenon of radio-resistance can affect the response to neoadjuvant chemoradiation (200), bringing more unfavorable treatment results in multiple cancers (201, 202), with ESC being no exception. The stemness of cancer cells was reported to participate in the occurrence mechanism of radio-resistance (203). Lowly expressed miR-99a-5p induced stemness maintenance of CSCs to resist radiotherapy in ESC. METTLl4 promoted DGCR8 to process pri-miR-99a-5p, causing mature miR-99a-5p to persist in ESCC. The overexpression of miR-99a-5p could weaken the capacity of DNA repair to increase the radiotherapy sensitivity of ESCC cells by preventing TRIB2 expression. As the downstream factor of miR-99a-5p, TRIB2 in turn negatively affected METTL14 expression. Mechanically, TRIB2 functioned as the bridge to join COP1 and METTL14, forming an ubiquitinated modification of METTL14 to facilitate its degradation. Taken together, METTL14, miR-99a-5p and TRIB2 constitute a special feedback loop to modulate the formation of radio-resistance in ESCC. Activators of METTL14 used in clinical treatment might improve the overall survival times of ESCC patients (137).

These explorations to m6A and ncRNAs deeper uncovered molecular mechanism of resistance of chemotherapy and radiotherapy, providing more possibilities of potential therapeutic targets. And the combination of inhibitors or activators of regulators and chemotherapy drug will ameliorate refractory of gastrointestinal cancers and improve prognosis of patients. But a part of regulators from m6A lack commercial inhibitors or activators. Corresponding side effect from molecular therapy still remains unknown. Due to diverse effects from m6A modification in cancers, only alteration m6A level does not necessarily achieve successfully. More precise target combined with m6A level changes need be considered.

## 6 Conclusion and prospects

In the past decades, m6A has been testified to occur in DNA, RNA and proteins, and implicated with the biological properties of multiple malignancies. The development of transcriptomics has brought the study of RNA to a climax, and a series of experiments gradually revealed the critical role of m6A on RNA in the regulation of post-transcriptional level. As the most common chemical modification type, m6A not only decorates mRNA but also appears in some non-coding RNAs, such as lncRNAs, miRNAs and circRNAs. The reversible effect of m6A relies on “writers”, “erasers” and “readers”.

These regulators can control the m6A level or directly affect ncRNA metabolism including biogenesis, location and degradation, resulting in positively or negatively altering the expression of ncRNAs. Thus, the abnormal expression of m6A and dysfunction of key regulators in these ncRNAs might contribute to the initiation and development of malignant tumors. Interestingly, non-coding RNAs can also exert an impact on the expression of regulator factors of m6A by indirectly modulating downstream targets or directly regulating particular factors of m6A, which has a close association with the biological function of GI cancers, including proliferation, invasion, migration, apoptosis, angiogenesis, and stemness. Nevertheless, the expression of ncRNAs and regulators would form a feedback loop to reciprocally mediate both sides, inducing chemotherapy resistance and causing dismal prognosis. Further studies on the interaction of m6A and ncRNAs can enrich the knowledge of the function of non-coding RNAs and dig up the comprehensive mechanism of the malignant phenotype of GI cancers.

As technologies and approaches of detecting m6A sites are gradually improved, we can progressively obtain more information on m6A alteration regarding higher-precision transcript segments, which can hopefully render m6A modifications more specific biomarkers in cancers. Regulators of m6A have not only proved as feasible in targeted treatment by numerous theories, but were initially developed by pharmaceutic or biotech companies to step into clinical therapy, with examples of METTL3 and METTL14 inhibitors, and so on. The utilization of potent inhibitors or activators to restore the normal expression level of m6A regulators can interrupt the oncogenic role of upstream or downstream targets, bringing a promising clinical prospect for patients, especially those with resistance to currently available treatments.

## Author contributions

YH and WG designed and guided the study. YX and XY wrote and edited the manuscript. YX helped with reference collection. All authors read and approved the final manuscript.
